# Detection of Tumor-Specific PTPmu in Gynecological Cancer and Patient Derived Xenografts

**DOI:** 10.3390/diagnostics11020181

**Published:** 2021-01-27

**Authors:** Jason Vincent, Sonya E. L. Craig, Mette L. Johansen, Jyosthna Narla, Stefanie Avril, Analisa DiFeo, Susann M. Brady-Kalnay

**Affiliations:** 1 Department of Molecular Biology and Microbiology, Case Western Reserve University, Cleveland, OH 44106, USA; jason.vincent@case.edu (J.V.); sonya.ensslen@case.edu (S.E.L.C.); mette.johansen@case.edu (M.L.J.); 2 Department of Pathology, Alexian Brothers Hospital, San Jose, CA 95116, USA; jyothsnie@yahoo.com; 3 Department of Pathology and the Case Comprehensive Cancer Center, Case Western Reserve University, Cleveland, OH 44106, USA; stefanie.avril@case.edu; 4 Department of Pathology and Department of Obstetrics & Gynecology, University of Michigan, Ann Arbor, MI 48109, USA; adifeo@med.umich.edu

**Keywords:** cancer, ovarian cancer, endometrial cancer, biomarker, protein tyrosine phosphatase, PTP, cell adhesion molecule, tumor microenvironment

## Abstract

Background: We developed a fluorophore-conjugated peptide agent, SBK4, that detects a tumor-specific proteolyzed form of the cell adhesion molecule, PTPmu, found in the tumor microenvironment. We previously demonstrated its tissue specific distribution in high-grade brain tumors. To extend those studies to other aggressive solid tumor types, we assessed the tissue distribution of PTPmu/SBK4 in a set of matched gynecologic cancer patient derived xenografts (PDXs) and primary patient tumors, as well as a limited cohort of tumors from gynecological cancer patients. PDXs isolated from the tissues of cancer patients have been shown to yield experimentally manipulatable models that replicate the clinical characteristics of individual patients’ tumors. In this study, gynecological cancer PDXs and patient biopsies were examined to determine if tumor-specific proteolyzed PTPmu was present. Methods: We used the peptide agent SBK4 conjugated to the fluorophore Texas Red (TR) to label tumor tissue microarrays (TMAs) containing patient and/or PDX samples from several high-grade gynecologic cancer types, and quantified the level of staining with Image J. In one TMA, we were able to directly compare the patient and the matched PDX tissue on the same slide. Results: While normal tissue had very little SBK4-TR staining, both primary tumor tissue and PDXs have higher labeling with SBK4-TR. Matched PDXs and patient samples from high-grade endometrial and ovarian cancers demonstrated higher levels of PTPmu by staining with SBK4 than normal tissue. Conclusion: In this sample set, all PDXs and high-grade ovarian cancer samples had increased labeling by SBK4-TR compared with the normal controls. Our results indicate that proteolyzed PTPmu and its novel peptide detection agent, SBK4, allow for the visualization of tumor-specific changes in cell adhesion molecules by tissue-based staining, providing a rationale for further development as an imaging agent in aggressive solid tumors, including gynecological cancers.

## 1. Introduction

There are many distinct gynecological cancers that can be divided into groups based on their tumor location, and are primarily ovarian, endometrial, and cervical. Ovarian cancer is the fifth leading cause of cancer-related deaths among women [1], and accounts for 2.3% of all cancer deaths in the U.S. [2]. While relatively rare, ovarian cancer was estimated to be diagnosed in 22,530 women in the U.S. in 2019, and its poor outcome is demonstrated by its low five-year survival rate of about 47.6% (seer.Cancer.gov), [2]. Uterine or endometrial cancer, on the other hand, is far more prevalent, with approximately 61,880 cases estimated to have been diagnosed in 2019. Uterine cancer has a much higher five-year relative survival rate of 81.2%, and accounts for only 2% of all cancer-related deaths [3]. Cervical cancer is the most rare of the three gynecologic tumor types, with 13,170 estimated new cases diagnosed in 2019 in the U.S., and a 65.8% five-year relative survival rate [4]. In the U.S., women have a 1.3, 3.1, and 0.6% lifetime risk of developing ovarian, uterine, and cervical cancer, respectively.

In recent years, patient derived xenograft (PDX) models have been developed to preserve original tumor characteristics, including the heterogeneous histology, clinical bio-markers, malignant phenotypes, and genotypes. Each PDX reflects the pathology of its original patient, while the cohort of patient PDXs represent the diversity of the human patient populations. Many currently available ovarian cancer cell lines do not genetically represent primary patient tumors, therefore our team generated a panel of primary patient PDXs to test the biomarkers in clinically relevant samples. We have demonstrated that several of these PDX models mimic the chemotherapy response of the patients from which they are derived [5].

The metastatic process includes the loss of contact inhibition mediated by cell–cell adhesion. Loss of the cellular signals needed to regulate proliferation and to maintain a cell in its current location results in the promotion of tumor cell migration. Cell adhesion molecules (CAMs) are transmembrane glycoproteins that mediate binding between CAMs on adjacent cells, and transduce these cell–cell signals into the cell via the cytoskeleton. In normal cells, full-length receptor protein tyrosine phosphatase PTPµ (PTPmu), like similar CAMs, mediates cell–cell adhesion. We previously hypothesized that the proteolysis of CAM extracellular domains promotes tumorigenesis [6,7]. We found evidence for the proteolysis of the homophilic binding CAM PTPµ into fragments in the tumor microenvironment of aggressive brain tumors, high-grade gliomas or glioblastomas [8,9,10], whereas higher levels of full-length PTPµ are expressed in normal and low-grade glioma tissue [11].

We hypothesized that the extracellular cleaved fragment of PTPµ is a biomarker of the tumor microenvironment, and developed peptide agents, SBK2 and SBK4, to bind to this fragment [12]. When conjugated to various fluorophores, in xenograft models of human glioblastoma (GB) in mice, both SBK2 and SBK4 detected tumors [12] and labeled 99% of GB cells when injected intravenously [13]. When conjugated to gadolinium chelators for use in MRI, we observed more extensive labeling of an invasive tumor and more sustained tumor binding than conventional contrast agents with the SBK2 agent [14,15,16]. We also tested the efficacy of the SBK4 agent conjugated to Texas Red (TR) as a one-step molecular diagnostic in human glioma tissue and found that it detected aggressive tumors [17], suggesting that adoption of the use of SBK4-TR in patient biopsy tissue could help stratify patient risk. In this study we set out to evaluate the efficacy of the SBK4-TR agent as a biomarker of other solid tumors, especially aggressive, invasive, and metastatic cancers. We selected gynecological cancers because they are difficult to detect and treat. Ovarian cancer is of high interest because of its poor overall survival.

## 2. Materials and Methods

### 2.1. Study Ethics and Patient Information

Patient samples were collected under two protocols approved by the University Hospitals Institutional Review Board (IRB). IRB protocol 1 included the prospective collection of discarded tissue and the generation of patient-derived xenografts with written informed consent obtained from the study subjects (PI: DiFeo). IRB protocol 2 included retrospective collection of archival discarded tissue samples with waived consent, including tumor and adjacent normal tissue (PI: Avril). As part of these IRB protocols, clinical and pathological data were gathered for some patients, and included age at diagnosis, race, tumor stage and grade, histological type, and overall survival.

### 2.2. Reagents

The SBK4 peptide, GIDVRDAPLKEIKVTSSR, used for the tissue staining, was synthesized on a CS Bio CS336X Synthesizer using Fmoc-protected amino acids and standard methods following previously described methods [12]. The *N*-terminal glycine of SBK4 peptide was coupled to Texas Red (TR; Molecular Probes Inc., Eugene, OR, USA) as described [12], to make the fluorescent agent. Anti-p53 Monoclonal antibodies (Biocare Medical, Pacheco, CA, USA) were used to stain the tissue sections to indicate nuclear staining and overexpression of p53 typical in high-grade serous ovarian carcinoma.

### 2.3. Biomarker Labeling of Human Gynecologic Tissue

All of the samples used for this study were obtained from the DiFeo and Avril labs. The Avril group generated tissue microarrays (TMAs) of several gynecologic cancers to facilitate screening a large number of patient tumor tissues. The DiFeo lab generated patient derived xenografts (PDXs) models from patient samples. To generate these PDX models, tumors and/or ascites were removed from patients and ~2 mm tumor implants were grafted subcutaneously, intraperitoneally, or into the ovarian bursa of Athymic Nude (nu/nu) mice and propagated. Sixty percent of the grafted tumors grew. Once tumors were detected, tumor volumes were measured weekly and were excised after they reached ~1000 mm^3^. Tumors were then (a) re-implanted into another nu/nu mouse, (b) processed using IHC analysis to compare the pathology to the original patient tumor, (c) processed for exome sequencing, and lastly (d) frozen in freezing media for future drug studies. Within this cohort of samples, we had tumors that represented several clinically distinct features including primary, metastatic, ascites, recurrent, and platinum-resistant tumors.

The DiFeo and Avril groups also generated TMAs that included tissue cores from PDX tumors and matched the patient primary tumors. Additional individual tumor and normal tissue samples were stained to increase the sample size. Together, these TMAs and individual specimens represented samples from 67 patients (43 with gynecologic cancer, 22 normal gynecologic tissues from patients without cancer, and 2 patients with both gynecologic cancer tissue and normal control tissue samples collected). The following gynecologic cancer types from distinct patients were represented in the dataset: endometrial clear cell carcinoma (*n* = 3), endometrial endometrioid carcinoma (*n* = 18), endometrial high-grade serous carcinoma (HG-EMCA; *n* = 6), ovarian high-grade serous carcinoma (HG-SOC; *n* = 16), and ovarian endometrioid carcinoma (*n* = 2). For the total PDX samples, there were *n* = 4 endometrioid endometrial carcinoma, *n* = 3 HG-EMCA, and *n* = 6 HG-SOC. The limited cohort with matched PDX-patient samples consisted of the following: *n* = 3 endometrial endometrioid carcinoma, *n* = 2 HG-EMCA, *n* = 2 ovarian endometrioid carcinoma, *n* = 1 uterine leiomyosarcoma, *n* = 1 endometrial undifferentiated carcinoma, *n* = 1 undifferentiated uterine sarcoma, *n* = 1 neuroendocrine tumor of the uterus, *n* = 1 ovarian carcinosarcoma, and *n* = 6 HG-SOC. Normal tissue samples came from 22 patients, and included normal endometrium (*n =* 11), normal ovary (*n =* 8), normal fallopian tube (*n =* 20), and normal cervix (*n* = 6).

Tissue staining with SBK4-TR was previously described [12] and is summarized briefly below. Positive controls (GBM) and negative controls (Epilepsy) were tested with the TMAs or individual slides. Tumor samples were obtained formalin-fixed and paraffin-embedded tissue. Prior to staining, the TMAs or slides were deparaffinized and blocked with 2% goat serum in phosphate buffered saline (PBS) for 20 min at room temperature (RT). The samples were then incubated with the SBK4-TR agent diluted in 2% goat serum in PBS at RT for 1 h in the dark. Following a PBS rinse, the TMAs or slides were cover-slipped with a Vectashield Hard Set Mounting Medium (Vector Laboratories, Inc., Burlingame, CA, USA) and were imaged on a Hamamatsu Nanozoomer S60 slide scanner (Bridgewater, NJ, USA). p53 immunohistochemistry was performed with antibodies from Biocare Medical, Pacheco, CA, USA.

Tissue staining with SBK4-TR was quantified using Image J and normalized to unit area of the region of interest. Student’s T-tests were conducted to determine statistical significance. In all cases, *p*-values < 0.05 were considered statistically significant. Where indicated, power analyses were performed using the statistical software R v4.0.3 from the R Foundation for Statistical Computing, Vienna, Austria (URL: https://www.R-project.org/), and the package MESS: Miscellaneous Esoteric Statistical Scripts by Claus Thorn Ekstrom (2020), R package version 0.5.7 (https://CRAN.R-project.org/package=MESS).

## 3. Results

### 3.1. Patient Derived Xenografts and Their Matched Patient Tumor Tissue Label with SBK4-TR

**Figure 1:**
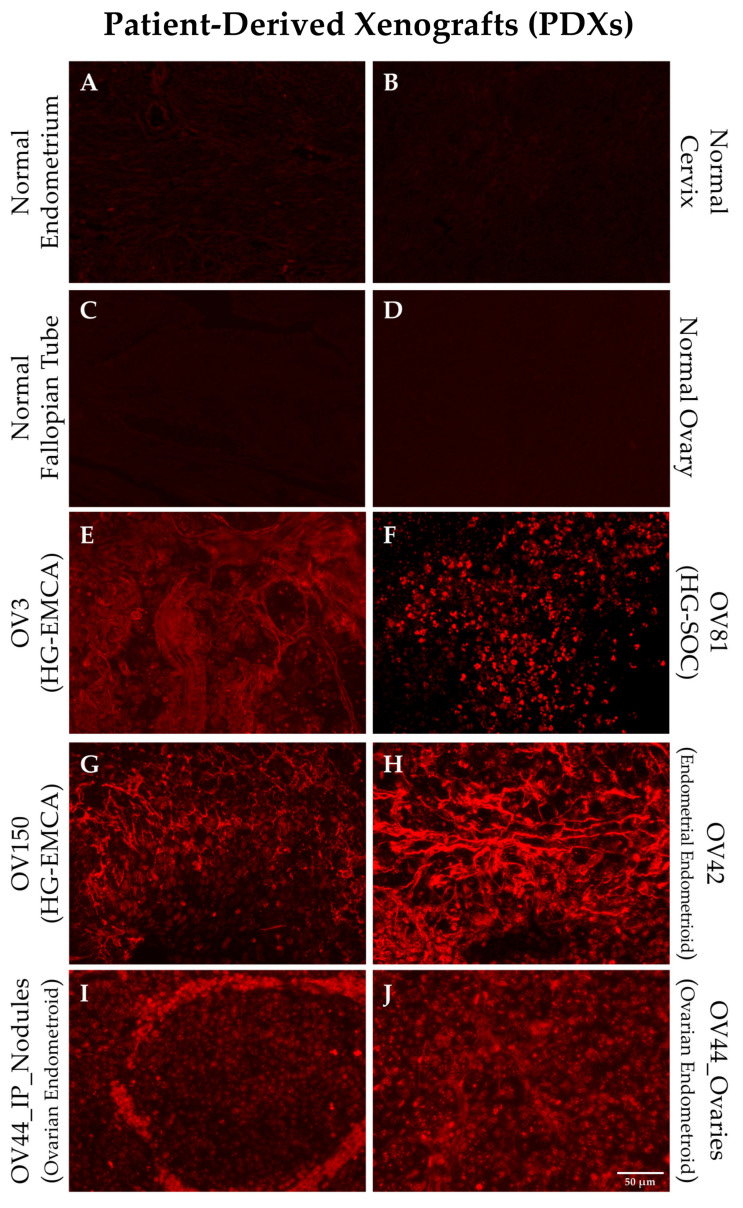
Staining of individual patient derived xenograft (PDX) tissue with SBK4-TR illustrate the high level of SBK4-TR staining intensity for different gynecological cancer PDXs (**E**–**J**) compared to normal controls (**A**–**D**). All of the samples had a higher level of staining than normal uterine, fallopian, ovarian, or cervical tissue. Panel **I** shows a metastasis from the primary tumor in panel **J**. Scale bar = 50 µm.

We generated a series of PDX models using tissue samples from gynecological oncology patients [5]. Individual tumor samples were obtained from the distinct PDX models and stained with the SBK4-TR agent to investigate whether the PTPµ biomarker was present. Figure 1 shows staining of PDX samples derived from HG-EMCA, ovarian endometrioid carcinoma, HG-SOC, and endometrial endometroid carcinoma (Figure 1). Notably, one ovarian endometrioid PDX grew in the ovary and metastasized to the intraperitoneal lining (Figure 1I,J). While normal tissue did not stain with SBK4-TR, all PDX tumors were highly labeled with SBK4-TR.

**Figure 2:**
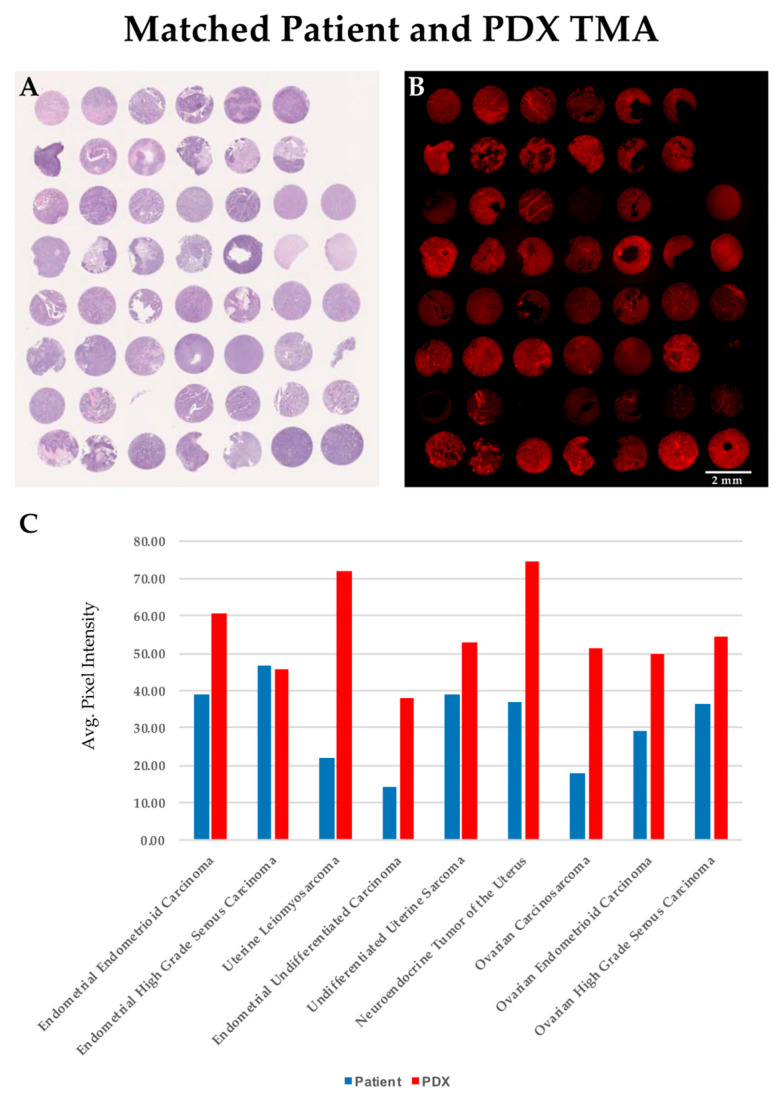
Comparison of SBK4-TR staining of matched PDX and patient samples indicates a high level of SBK4-TR labeling for all cancer tissues. Average staining data are shown in panel (**C**). Comparison of adjacent PDX/patient matched tissue microarrays (TMAs) stained for hematoxylin and eosin (**A**), and SBK4-TR (**B**), indicate the high level of overall staining. Scale bar = 2 mm. We calculated the average SBK4-TR staining of patient normal, patient tumor and PDX samples for nine tumor types that had both patient and matched PDX tumor tissue (**C**). Plotting of the average values obtained for the tissue biopsy cores for the patient and PDX tumors illustrates the overall high level of staining intensity of patient and matched PDX samples (**C**).

We generated a TMA to directly compare the level of SBK4-TR staining of individual patient tumor samples with their exact matched PDX tumors. Individual tissue punches from the matched PDX and patient tissue sections were stained by hematoxylin and eosin (Figure 2A) or labeled by the SBK4-TR agent, as shown in Figure 2B. Note that sometimes tissue is lost from a particular TMA slide during washes, but is present in the other slide. To compare the relative level of SBK4-TR staining, Image J software was used to highlight the entire tissue section and calculate an absolute value for the SBK4-TR signal normalized to unit area. The average values for each type of tissue, whether from TMA or tissue sections, were determined and plotted (Figure 2C). SBK4-TR staining data were obtained for nine different gynecological tumor types with matched patient and PDX samples. The SBK4-TR agent detected the PTPµ biomarker in all of these matched samples (Figure 2C). The process of PDX tumor generation tends to select for the most aggressive and tumorigenic cells. Thus, it was notable that the highest levels of the PTPµ biomarker were present in the PDX tumors.

### 3.2. Gynecological Cancer Tissue Staining for the PTPµ Biomarker

**Figure 3:**
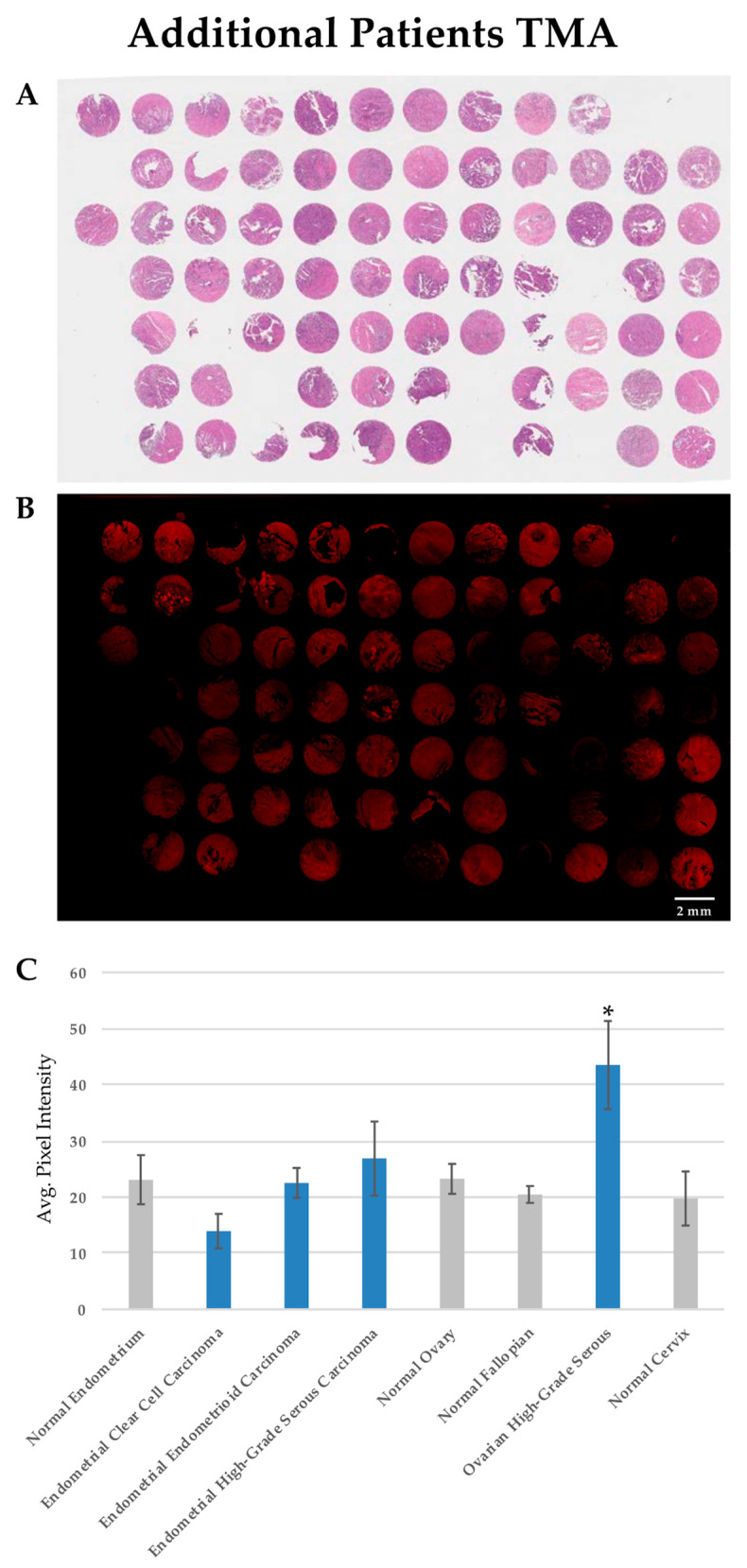
SBK4-TR staining of patient gynecologic cancer tissue section TMAs. Staining of adjacent TMAs with hematoxylin and eosin (**A**), and SBK4-TR (**B**). SBK4-TR staining shows various levels of staining intensity (**B**). Scale bar = 2 mm. The mean SBK4-TR staining intensity is significantly higher in high grade serous ovarian cancer compared with normal fallopian tube (* *p* = 0.012) or normal ovary (* *p* = 0.028). The other cancer types are similar to the controls (**C**). Mean and standard error (SE) are shown for the indicated tissue types.

For this study, individual normal and tumor samples were obtained from 67 patients, including 43 patients with gynecological cancers, 22 normal gynecologic tissues from patients without cancer, and 2 patients with both gynecologic cancer and adjacent normal tissue samples. The majority of these samples were examined for the PTPµ biomarker in TMAs, including the 20 specimens used for the derivation of the PDX samples. The remaining samples were examined as tumor tissue specimens on conventional slides. The clinicopathological characteristics of the patients from whom the PDX tissue was obtained are summarized in Table 1, while all patients are summarized in Table 2. Hematoxylin and eosin staining is shown for tissue architecture (Figure 3A). SBK4-TR stained most of the tumor tissue sections, although the intensity of staining varied between individual samples (see Figure 3B for an example). SBK4-TR signal intensities were determined as described above, and the values from the samples in both the TMAs and tissue sections are plotted in Figure 3C where the mean SBK4-TR signal intensity in high-grade serous ovarian cancer (HG-SOC) was statistically greater than that in both normal ovary (*p* = 0.028) and normal fallopian tube (*p* = 0.012), indicating higher levels of the PTPµ biomarker in this aggressive tumor type. Based on the HG-SOC and normal fallopian tube sample sizes, along with the mean and standard deviations of each, the difference in mean SBK4-TR staining was determined with 76% power. The other tumor tissue types, including endometrial endometrioid carcinoma or endometrial clear cell carcinoma, were similar to the normal endometrium control tissue. Some HG-EMCA samples had higher levels than the controls, but were more variable and did not reach statistical significance.

### 3.3. PTPµ Biomarker Staining is High in Patients with HG-SOC

**Figure 4:**
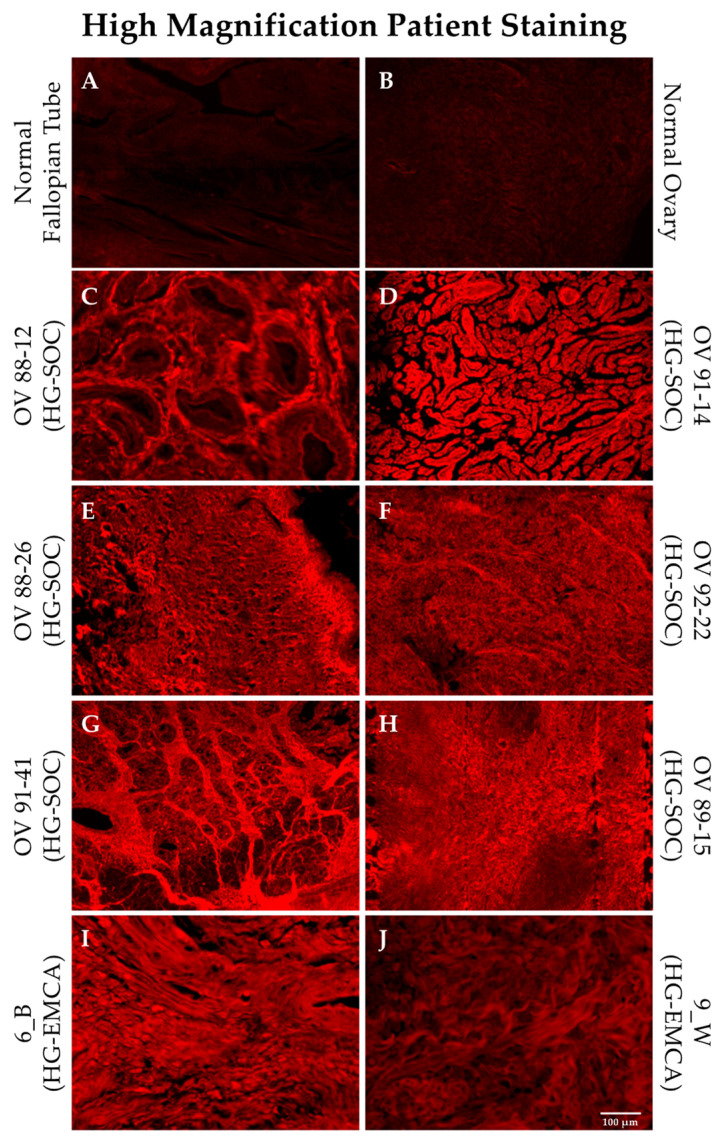
SBK4-TR staining of HG-SOC and HG-EMCA tissue. Representative tissue sections from patients with HG-SOC (panels **C**–**H**) and HG-EMCA (panels **I**,**J**) are shown stained with SBK4-TR and are compared with normal tissue sections stained with SBK4-TR (panels **A**,**B**). Scale bar = 100 µm.

HG-SOC and HG-EMCA tissues stained with SBK4-TR were more closely examined using high magnification in order to determine whether the PTPµ biomarker was localized in any particular manner. Normal fallopian tube and ovary show very low levels of SBK4-TR staining (Figure 4, panels A and B). Examples of HG-SOC are shown in Figure 4, panels C–H, alongside the control tissues. Representative sections from patients with HG-EMCA are also shown (Figure 4, panels I, J), with similar levels of SBK4-TR staining intensity, although this tissue staining was more variable.

### 3.4. SBK4-TR Labels Metastatic Nodules in HG-SOC

**Figure 5:**
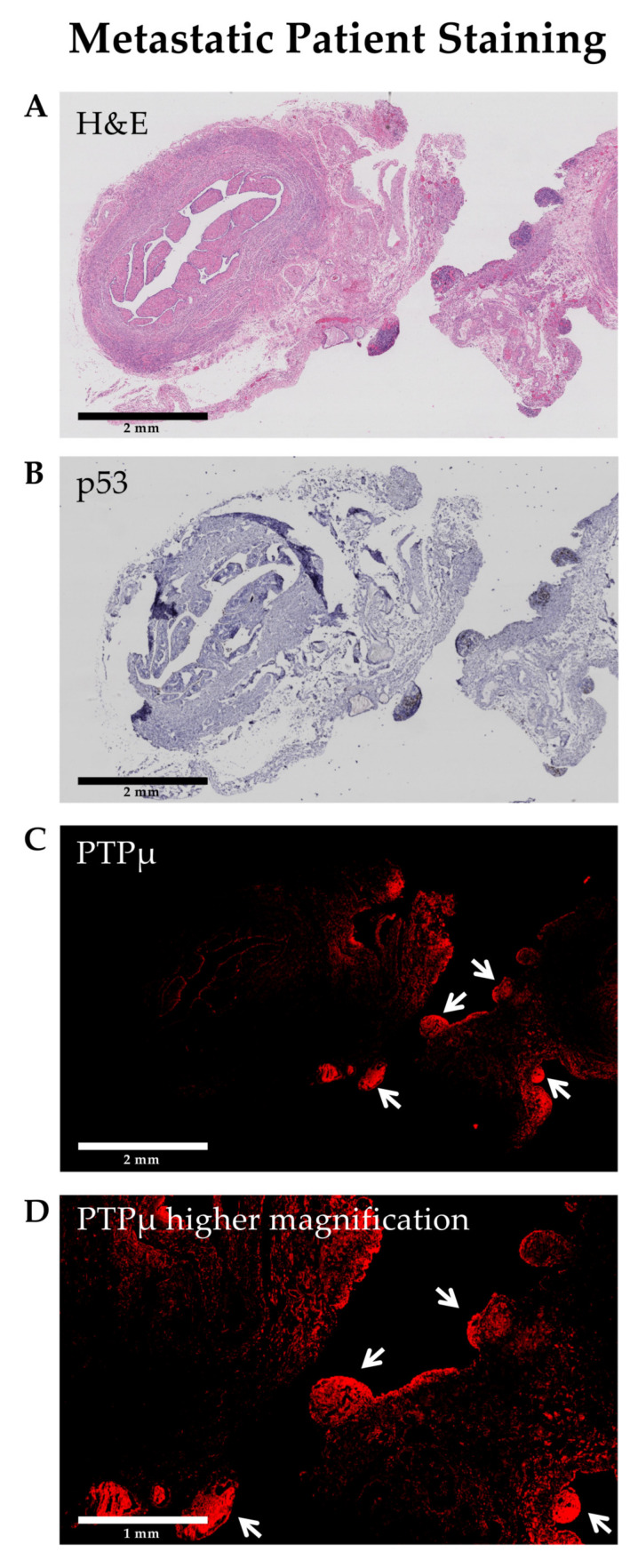
Invasive and metastatic high grade serous ovarian cancer nodules (arrows) are positive for both p53 and the PTPµ biomarker. Staining of adjacent tissue sections for hematoxylin and eosin (**A**), p53 (**B**), and SBK4-TR (**C**) demonstrates that areas of tumor growth are labeled by both p53 antibodies (mutated pattern) and SBK4-TR. Scale bars = 2 mm. A higher magnification image from a region in panel (**C**) is shown in panel (**D**). Scale bar = 1 mm.

Several markers are commonly used to identify HG-SOC in pathology sections, however, mutated p53 (strong overexpression or complete absence) is considered a hallmark that defines high grade serous ovarian cancer [18]. To examine the location of the PTPµ biomarker in the context of p53, adjacent HG-SOC sections were stained for each marker and were compared (Figure 5). The HG-SOC example shown in Figure 5 includes p53 positive invasive and metastatic nodules on otherwise normal fallopian tube tissue (areas of darker H&E staining in panel A, and darker blue staining in panel B). As shown in Figure 5C, these nodules also show intense SBK4-TR staining.

## 4. Discussion

These results expand on our earlier observations [5], demonstrating that gynecological cancer PDX tissue serves as a useful surrogate to match patient tissue samples. Here, we show that the PTPµ biomarker, as revealed by SBK4-TR staining, was elevated in PDX and matched patient tumor samples. Importantly, we found significantly higher levels of the PTPµ biomarker in HG-SOC relative to normal fallopian tube and normal ovary. We also investigated four other gynecological tumors types, and found variable levels of SBK4-TR staining. Only HG-EMCA showed higher levels in some samples, but the other tumor types did not differ significantly from that in the corresponding normal tissues. HG-SOC is an aggressive, invasive, and metastatic tumor type with very poor outcomes. Characteristic of a type II ovarian carcinoma is its rapid spread throughout the body. HG-SOC cells usually originate in the fallopian tube, but may be primarily ovarian or peritoneal in a final metastatic location [19]. Its aggressive nature is supported by the fact that HG-SOC is responsible for 70–80% of all ovarian cancer deaths [1]. Genomic studies have identified a high level of genomic instability in patients with HG-SOC, including frequent TP53 mutations [1,19]. A mutation and thus marker shared by both gliomas and HG-SOC is p53.

We have previously observed that SBK4-TR preferentially labels the margins of invasive brain tumors [12]. In this manuscript, we made a similar observation with SBK4-TR staining metastatic nodules in both PDX tumors (Figure 1I) and patient tissue biopsies (Figure 5). Furthermore, both SBK4-TR and mutated p53, a hallmark of high-grade serous tumor cells, labeled the same hypertrophic nodules in an invasive and metastatic HG-SOC sample. These results suggest that PTPµ agents may be utilized for visualizing invasive and metastatic tumors in different applications, including fluorescence-guided resection during abdominal surgeries.

Proteolysis of cell–cell adhesion molecules occurs preferentially in cancer tissue [6,7] and we propose that it is one mechanism for promoting loss of contact inhibition of growth and migration in cancer cells [6]. We have extensively studied the proteolysis of the cell–cell adhesion molecule PTPµ in gliomas [6,7,8,10,11,13,14,15,16,17]. Unlike the normal full-length forms of cell adhesion molecules, proteolytically cleaved fragments can have oncogenic properties [8,10]. For example, cleavage of PTPµ has the effect of dissociating adhesion from phosphatase activity as well as altering the subcellular localization of the phosphatase enzyme. The reduction of full-length PTPµ [11] and the presence of PTPµ fragments contribute to tumor cell growth and migration [8]. The stimuli for proteolysis include growth factor stimulation and calcium influx, which are implicated in tumor progression [8,10].

## 5. Conclusions

Similar to our findings in aggressive glioblastoma tumors, the proteolysis of cell adhesion molecules occurs in aggressive gynecological cancer PDXs and patient samples, as demonstrated by staining with the PTPµ proteolytic fragment detection agent, SBK4-TR. Furthermore, both SBK4-TR and mutated p53, a hallmark of high-grade serous tumor cells, are co-localized in metastatic HG-SOC. The high levels of the PTPµ biomarker in primary and metastatic HG-SOC warrant further investigation as an imaging agent for gynecological tumors.

## Figures and Tables

**Table 1:** Clinicopathological characteristics of patients from whom PDXs are derived.

Histology	Endometrial Endometrioid Carcinoma	HG-EMCA	Uterine Leiomyo-Sarcoma	Endometrial Undifferentiated Carcinoma	Undifferentiated Uterine Sarcoma	Neuro-Endocrine Tumor of the Uterus	Ovarian Carcino-Sarcoma	HG-SOC	Ovarian Endometroid Carcinoma
**Number of Patients**	4	3	1	1	1	1	1	6	2
**Tumor Grade (%)**									
FIGO grade 1	1 (25)								
FIGO grade 2	1 (25)								
FIGO grade 3	2 (50)	1 (33.3)	1 (100)	1 (100)	1 (100)	1 (100)	1 (100)	5 (83.3)	1 (50)
High grade		2 (66.7)						1 (16.7)	1 (50)
**Tumor Stage (%)**					N/A				
I	1 (25)					1 (100)			1 (50)
II									1 (50)
III	2 (50)						1 (100)	3 (50)	
IV	1 (25)	3 (100)	1 (100)	1 (100)				3 (50)	
**Number African American Patients (%)**	1 (25)	3 (100)	0		0	1 (100)	0	0	1 (50)
**Number Caucasian Patients (%)**	3 (75)	0	1 (100)		1 (100)	0	1 (100)	6 (100)	1 (50)
**Mean Age**	53.5	70.3	47	54	67	62	82	54.0	60
**Average SBK4 staining (SE)**	44.5 (5.6)	41.1 (6.43)	71.91 (N/A)	37.7 (N/A)	52.9 (6.0)	74.8 (N/A)	51.2 (N/A)	54.51 (6.0)	49.8 (5.2)

**Table 2:** Clinicopathological characteristics of gynecological cancer patient samples.

Histological Type	Endometrial Clear Cell Carcinoma	Endometrial Endometrioid Carcinoma	HG-EMCA	HG-SOC	Ovarian Endometroid Carcinoma
**Number of Patients**	3	18	6	16	2
**Tumor Grade (%)**					
FIGO grade 1		5 (27.8)			0
FIGO grade 2		5 (27.8)			0
FIGO grade 3		8 (44.4)			2 (100)
High grade	3 (100)		6 (100)	16 (100)	
**Tumor Stage (ratio)**					
I	2 (2/3)	9 (9/17)	2 (2/6)	0	1 (1/2)
II	0	1 (1/17)	0	0	0
III	1 (1/3)	4 (4/17)	1 (1/6)	12 (12/16)	1 (1/2)
IV	0	3 (3/17)	3 (3/6)	4 (4/16)	0
**Number African American Patients (ratio)**	3 (3/3)	7 (7/16)	5 (5/6)	0 (0/9)	1 (1/2)
**Number Caucasian Patients (ratio)**	0 (0/3)	9 (9/16)	1 (1/6)	9 (9/9)	1 (1/2)
**Mean Age (SD)**	73.3 (13.1)	57.4 (10.5)	68.2 (9.6)	56.9 (8.7)	60 (2.82)
**Average SBK4 staining (SE)**	13.9 (3.1)	23.4 (2.3)	26.9 (6.6)	44.0 (8.05)	29.0 (1.0)

## Data Availability

Data is contained within the article.
